# Relationship Between Anemia and Oral Lichen Planus: New Therapeutic Perspectives Based on Anemia Management—A Systematic Review and Meta-Analysis

**DOI:** 10.3390/jcm15020581

**Published:** 2026-01-11

**Authors:** Sonia Egido-Moreno, Joan Valls-Roca-Umbert, Mayra Schemel-Suárez, August Vidal-Bel, Andrés Blanco-Carrión, José López-López

**Affiliations:** 1 School of Medicine and Dentistry, University of Santiago de Compostela, 15782 Santiago de Compostela, A Coruña, Spain; andres.blanco@usc.es; 2 Department of Odontostomatology, Faculty of Medicine and Health Sciences (Dentistry), University of Barcelona, 08907 L’Hospitalet de Llobregat, Barcelona, Spain; joan.valls@yahoo.es (J.V.-R.-U.);; 3 Pathological Anatomy Department, University Hospital o Bellvitge, 08970 L’Hospitalet de Llobregat, Barcelona, Spain; augustvidalbel@gmail.com

**Keywords:** oral lichen planus, anemia, iron deficiency, vitamin B12 deficiency, folic acid deficiency

## Abstract

**Background/Objectives**: Anemia is a multifactorial condition influenced by nutritional deficiencies, chronic diseases, and inflammatory processes. These factors not only contribute to anemia but may also exacerbate oral conditions such as Oral Lichen Planus (OLP) by impairing epithelial integrity and immune function. By synthesizing published studies, this review seeks to clarify whether anemia is associated with OLP and to highlight biological mechanisms common to both conditions that could be relevant for future therapeutic development. **Methods**: A comprehensive literature search was conducted across the selected electronic databases: Medline/Pubmed, Scopus, and Cochrane. Methodological quality and potential bias of the included studies were evaluated using the Newcastle–Ottawa Scale (NOS), while the overall certainty of the evidence was appraised according to the Grades of Recommendation, Assessment, Development and Evaluation (GRADE) framework. Forest plots were generated using the Cochrane RevMan software to evaluate and visually summarize the results of the included studies. **Results**: Application of the search strategy resulted in the identification of 549 articles; after applying exclusion and inclusion criteria, 11 papers were selected. The prevalence of anemia, iron deficiency, and folic acid deficiency was significantly increased in the study population (*p* < 0.05); whereas hemoglobin deficiency was observed exclusively in women with statistical significance (*p* < 0.00001), driven by a single large study. **Conclusions**: Patients with OLP show a higher prevalence of anemia and deficiencies in key hematologic micronutrients such as vitamin B12, folic acid, and iron. Routine laboratory evaluation is recommended to detect and manage these systemic alterations. In addition to corticosteroid therapy, micronutrient supplementation may serve as a useful complementary treatment approach.

## 1. Introduction

Due to its diverse etiologies, anemia is considered a major global health challenge. According to the World Health Organization (WHO), it is defined by hemoglobin (Hb) levels below 13.0 g/dL in men and 12.0 g/dL in women [1].

The etiology is complex and often multifactorial. Nutritional deficiencies—particularly of iron, folate, and vitamin B12—remain the most prevalent. These insufficiencies commonly arise from inadequate intake, chronic blood loss, or impaired intestinal absorption. Infectious and parasitic diseases, such as malaria and helminth infections, contribute to anemia through red blood cell destruction and blood loss, while chronic infections like HIV and tuberculosis induce inflammation-mediated anemia via increased hepcidin levels and disrupted iron metabolism. In addition, hereditary Hb disorders, including thalassemia and sickle cell disease, represent significant contributors. Physiological conditions related to high metabolic demands during pregnancy, childhood, and adolescence increase susceptibility to anemia [2].

Iron deficiency anemia is characterized by diminished iron availability, which limits Hb formation and leads to the production of small, lightly pigmented erythrocytes. Due to inadequate dietary iron intake, ongoing losses (such as heavy menstruation or parasitic infections), and reduced absorption caused by infections or inflammation, which increases hepcidin and inhibits iron release. In addition, deficiencies in other micronutrients such as vitamin A, vitamin B12, and folate also play a significant role in the causes of anemia. Vitamin A deficiency, although in itself is not classified as anemia, it can indirectly contribute to anemia through several mechanisms: it affects erythropoiesis, impairs the release of iron from stores, and compromises immunity, increasing susceptibility to infections that exacerbate anemia. Meanwhile, deficiencies in vitamin B12 and folate disrupt DNA synthesis and the proliferation of red blood cell progenitor cells, resulting in megaloblastic anemia, while low folate levels can also decrease the lifespan of erythrocytes [2].

Causes of anemia can also originate from the gastrointestinal tract. Impaired intestinal absorption of essential nutrients, such as iron and vitamins, that is, from malabsorption [3]. Chronic occult blood loss from the gastrointestinal tract, such as from ulcers, angiodysplasia, or gastrointestinal malignancies, which can also lead to anemia [4]. In addition, anemia may also result from intrinsic factor deficiency. In this condition, commonly associated with autoimmune atrophic gastritis and pernicious anemia, the absence of intrinsic factor prevents the binding of vitamin B12 and the formation of the vitamin B12–intrinsic factor complex, which is required for absorption in the terminal ileum. Consequently, vitamin B12 deficiency develops, leading to megaloblastic anemia as another gastrointestinal-related mechanism of anemia [3].

Anemia is usually associated with nonspecific clinical manifestations resulting from reduced oxygen-carrying capacity of the blood. Typical symptoms include fatigue, weakness, pallor, reduced exercise tolerance, dizziness, and shortness of breath, while more severe or prolonged anemia may lead to palpitations and tachycardia. The consequences of anemia depend on its severity, duration, and underlying cause, and may include impaired physical and cognitive performance, decreased work capacity, and reduced quality of life. In vulnerable populations such as pregnant women and children, anemia has been associated with adverse outcomes, including impaired growth and development, whereas in adults and older individuals, it may exacerbate existing comorbidities and increase morbidity [1,2].

On the other hand, the deficiencies could also be in relationship with Oral Lichen Planus (OLP). OLP is a chronic inflammatory disorder affecting the oral mucosa and is characterised by a persistent or recurrent clinical course. It is considered an immune-mediated disease in which T lymphocytes play a central role in inducing basal keratinocyte damage and epithelial disruption [5,6]. Due to its chronicity, frequent symptoms such as pain or burning sensation, and the need for long-term monitoring, oral lichen planus has a significant impact on patients’ quality of life and represents a relevant condition in daily clinical practice [6].

Although OLP primarily affects the oral mucosa, it is increasingly recognized as a condition influenced by systemic factors. Previous research has reported associations between OLP and several comorbidities, including autoimmune, metabolic, and psychological disorders [7]. These findings support the concept that OLP should not be regarded solely as a local disease, but rather as a condition potentially modulated by the patient’s overall systemic and inflammatory status, including nutritional and hematological factors [8].

**Figure 1:**
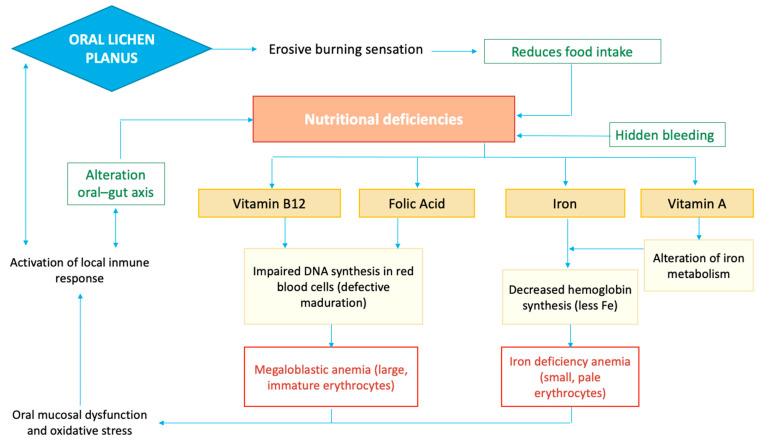
Flowchart representation of the mechanisms associated with Anemia and OLP.

Iron deficiency may compromise epithelial integrity and immune function in the oral mucosa, thereby contributing to the pathogenesis or severity of OLP, as iron is essential for epithelial cell turnover and effective immune responses [9]. Similarly, folate and vitamin B12 deficiencies may contribute to mucosal lesions, chronic inflammation, and impaired epithelial cell maturation, which may predispose to or aggravate OLP lesions [10]. Reduced levels of vitamin B12, frequently reported in symptomatic OLP patients, may further impair DNA synthesis and repair, contributing to epithelial dysregulation and sustained immune-mediated tissue damage [10,11]. Given the role of vitamin B12 in cellular metabolism and immune modulation, its insufficiency could facilitate disease persistence and potentially favor malignant transformation in susceptible lesions [11]. Likewise, studies suggest that vitamin A plays a role in maintaining epithelial integrity, and alterations in its local metabolism may contribute to increased mucosal vulnerability and inflammation in OLP [12]. Figure 1 summarizes the relationship between OLP, nutritional deficiencies, and anemia. It illustrates how OLP may contribute to iron and vitamin deficiencies because of mucosal alterations that contribute to reduced food intake. These deficiencies impair erythropoiesis, with iron deficiency leading to anemia.

Chronic inflammation and nutritional deficiencies represent common pathogenic mechanisms between anemia in older adults and OLP. This disease can have systemic repercussions via the oral–gut axis. Proinflammatory cytokines such as IL-6, TNF-α, and IFN-γ, which can compromise intestinal barrier function and promote a state of dysbiosis. These intestinal alterations may increase epithelial permeability and impair nutrient absorption, potentially leading to malabsorption and creating a systemic inflammatory loop that exacerbates oral pathology [13]. These effects, combined with potential deficiencies in iron, vitamin B12, or folate, contribute to the development of anemia of inflammation [14]. In the same way, in OLP, these T cell–mediated cytokines induce keratinocyte apoptosis and damage to the basal layer, while nutritional disorders may influence the progression and severity of the disease [15].

Although several studies have explored the relationship between OLP and anemia or related hematological alterations, the available evidence remains heterogeneous and, in some cases, contradictory [16]. As a result, there is currently no comprehensive synthesis clarifying whether anemia should be routinely considered in the clinical evaluation of patients with OLP or what clinical implications this association may have for patient management. Given the overlapping pathogenic mechanisms implicated in OLP and anemia, the present study aims to review the existing literature to examine the potential relationship between anemia and OLP and to explore possible therapeutic implications derived from their shared etiopathogenic pathways.

## 2. Materials and Methods

This systematic review was conducted in accordance with the Preferred Reporting Items for Systematic Reviews and Meta-Analyses (PRISMA) statement [17] (see Supplementary Material). A detailed review protocol defining the objectives, eligibility criteria, and methodological approach was developed a priori and registered in the International Prospective Register of Systematic Reviews (PROSPERO) under the registration number CRD420251231553.

### 2.1. Focused Question

Is there an association between OLP and anemia in adult patients?

### 2.2. PICO Question

(P) Population: Adult patients diagnosed with OLP and adult individuals without OLP;
(I/E) Intervention/event: Patients with OLP;
(C) Comparison: Patients without OLP;
(O): Outcome: Prevalence of anemia and/or hematological parameters related to anemia, including hemoglobin levels and biomarkers of iron metabolism, vitamin B12, and folic acid.

### 2.3. Eligibility Criteria

Inclusion criteria: observational studies (cohort, case–control or cross-sectional studies); Studies involving adult patients; All studies will be included with no restrictions regarding date or language; Assessment of anemia based in blood samples; Reporting of at least one analytical parameter related to anemia, such as hemoglobin, iron metabolism parameters, vitamin B12, or folic acid.

Exclusion criteria: case series without control group; studies involving pediatric population.

### 2.4. Search Strategy

A literature search was performed without time restrictions up to 12 November 2025. Relevant publications were retrieved from Medline/PubMed, Scopus, and the Cochrane Library.

The terms used for the search were as follow: (“oral lichen planus” [Mesh] AND “anemia” [Mesh]), (“oral lichen planus” [Mesh] AND (“hemoglobin” [Mesh] OR “hemoglobin deficiency” [All Fields])), (“oral lichen planus” [Mesh] AND (“iron” [Mesh] OR “iron deficiency” [All Fields])), (“oral lichen planus” [Mesh] AND (“vitamin B12” [Mesh] OR “vitamin B12 deficiency” [Mesh])), (“oral lichen planus” [Mesh] AND (“folic acid” OR “folic acid deficiency” [Mesh])), (“oral lichen planus” [Mesh]. The same combination of search terms was consistently employed in each database, minor modifications to the syntax were made as necessary to accommodate database-specific search interfaces.

### 2.5. Study Selection Process

Two authors (S.E.-M. and J.V.-R.) independently conducted the study selection process, beginning with the screening of titles and abstracts retrieved from the databases. Articles deemed eligible were subsequently reviewed in full text, and their reference lists were searched for further relevant publications. Disagreements were addressed by consensus with the involvement of two other investigators (A.B.-C. and J.L.-L.). Agreement between reviewers was quantified using Cohen’s kappa statistics for each database.

### 2.6. Data Extraction

Two investigators (S.E.-M. and J.V.-R.) independently collected study data following a predefined extraction protocol. Extracted variables comprised bibliographic information, study setting, methodological design, participant numbers in OLP and control groups, demographic data, and assessed analytical outcomes. Summary estimates were calculated using a random/fixed-effects meta-analytic approach.

### 2.7. Data Synthesis and Analysis

Results were quantitatively synthesized using a random/fixed-effects model. The principal effect size was the mean difference in anemia-related biomarkers between individuals with OLP and control subjects. Study estimates were combined using the inverse-variance method. Statistical heterogeneity was evaluated with the I^2^ statistic, considering a *p* value below 0.10 as indicative of significant heterogeneity. Interpretation of I^2^ values followed the guidance of the Cochrane Handbook, classifying heterogeneity as negligible (0–40%), moderate (30–60%), substantial (50–90%), or considerable (75–100%). Where sufficient data were available, subgroup analyses were performed. All meta-analyses were conducted using Review Manager 5.4 (RevMan) software, and outcomes with notable clinical relevance are explicitly reported in the Results section.

### 2.8. Risk of Bias

The Newcastle–Ottawa Scale (NOS) [18] was applied to assess the risk of bias and methodological rigor of the included observational studies. Evaluation was based on three core domains, namely participant selection, group comparability, and the determination of exposure or outcome.

Studies were classified according to the following criteria:

- Good quality: 3–4 stars in the selection domain, 1–2 stars in the comparability domain, and 2–3 stars in the exposure domain.
- Fair quality: 2 stars in the selection domain, 1–2 stars in the comparability domain, and 2–3 stars in the exposure domain.
- Poor quality: 0–1 star in the selection domain, 0 stars in the comparability domain, or 0–1 star in the exposure domain.

### 2.9. Grading of Recommendations Assessment, Development and Evaluation

The overall certainty of the evidence was evaluated using the GRADE framework [19]. This methodology assigns an initial confidence level according to study design and subsequently considers several factors, such as risk of bias, heterogeneity, indirectness, imprecision, publication bias, dose–response effects, residual confounding, and the magnitude of observed associations, to derive a final certainty rating. Evidence classified as moderate or high indicates a reasonable to strong level of confidence in the findings, whereas low or very low ratings reflect limited confidence in the estimated effects.

## 3. Results

### 3.1. Study Selection

**Figure 2:**
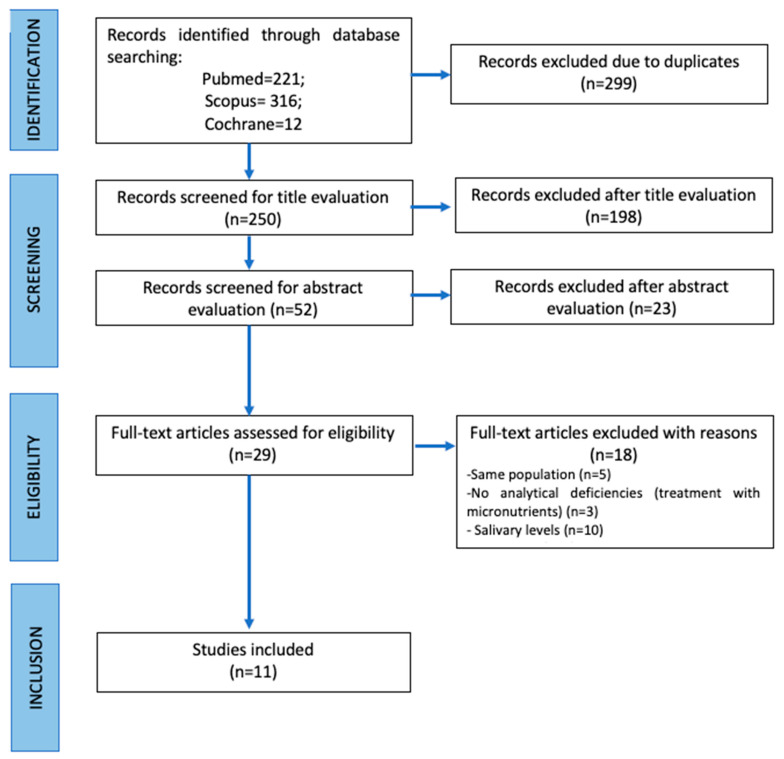
Flow diagram of the study selection process according to Cochrane guidelines.

A total of 549 publications were retrieved through the search process. Following duplicate removal, 299 records were screened. Of these, 198 were excluded after title and abstract review, resulting in 29 articles eligible for full-text evaluation. Eighteen were excluded, 5 articles studied the same population, 10 articles studied salivary levels instead of blood markers, and 5 articles used micronutrients as a treatment without specifying nutrient deficiencies. Finally, 11 studies were selected Figure 2. The Cohen kappa was 0.81 for Medline/Pubmed, 0.73 for Scopus, 1 for Cochrane Library.

### 3.2. Study Characteristics

All the studies included were in English. The characteristics of the studies are summarized in Table 1. The included articles were cross sectional case–control studies. Two studies were conducted in China [16,20], two in India [11,21], two in Taiwan [22,23], two in Iran [24,25], one in Thailand [10], one in Egypt [26], and one y Hungary [27]. In all included studies, the diagnosis of OLP was established using a combination of clinical assessment and histopathological confirmation Table 2. The population included were 1.264 patients with OLP, 1331 healthy patients. In the study by Wu et al. [22], patients were classified dichotomously into iron deficiency anemia (IDA) (n = 75) and non-IDA (n = 150) groups. This approach differs from most studies, which typically stratify patients according to the presence or absence of OLP rather than by anemia status. Finally, in another study the study group was patients with Potentially Malignant Disorders (n = 75). In reference to sex, nine articles [10,16,20,21,23,24,25,26,27] provide data separated by male and female groups. The study by Naik et al. [11] only reports sex data for the OLP group, while the study by Wu et al. [22] provides sex data for the IDA group (64 females and 11 males) and for the non-IDA group (28 females and 22 males). In the OLP group, there were 27.91% (n = 364) males and 72.09% (n = 94) females, and in the control group, 30.24% (n = 381) males and 69.76% (n = 879) females. Regarding age, eight studies [10,16,20,21,23,24,25,26,27] provide data separately by groups; The study of Sahebjamee et al. [24] did not provide data regarding age, while the study by Naik et al. [11] only reports data for the entire population, which was 38.5 years; and the study of Wu et al. [22] provides data according to whether they have IDA or not, with ages of 51.7 ± 14.1 and 52.4 ± 13.8, respectively. The mean age for OLP group was 52.47 ± 5.08 years and for the control group was 50.99 ± 6.7 years.

### 3.3. Risk of Bias and Grading of Recommendation

Table 3 presents an overview of the methodological quality and certainty of evidence of the included studies, as evaluated using the Newcastle–Ottawa Scale (NOS) [13] and the GRADE framework [14]. Most studies examining the association between hematinic deficiencies, blood biomarkers, and oral mucosal lesions are cross-sectional with defined case and control groups, with high NOS scores (≥7/9), indicating well-defined groups, objective measurements, and adequate comparability. According to GRADE, the evidence is moderate (Level 2/Grade B), supporting associations but not causal inference. The study of Yao et al. has a lower NOS score (5/9) and limited evidence (Level 3/Grade C), reflecting higher risk of bias and lower reliability.

### 3.4. Statistical Analysis

To visually summarize the included studies, forest plots were created for graphical representation.

**Figure 3:**
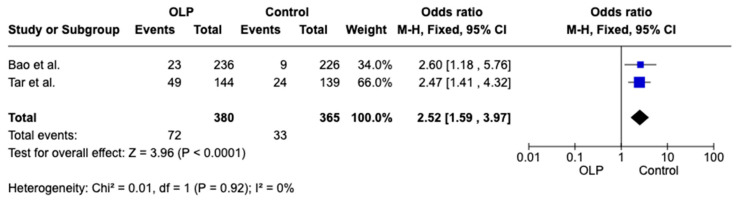
Forest plot showing the difference in anemia prevalence between OLP patients and healthy controls [16,27].

Two papers [16,27] of the included studies reported on the overall presence of anemia in patients with OLP and healthy controls, revealing a significant difference between the groups. Weighted Mean Difference (WMD): 2.52, 95% CI: −1.59 to 3.97, *p* = 0.02 and heterogeneity 0% *p* < 0.92, as shown in Figure 3.

**Figure 4:**
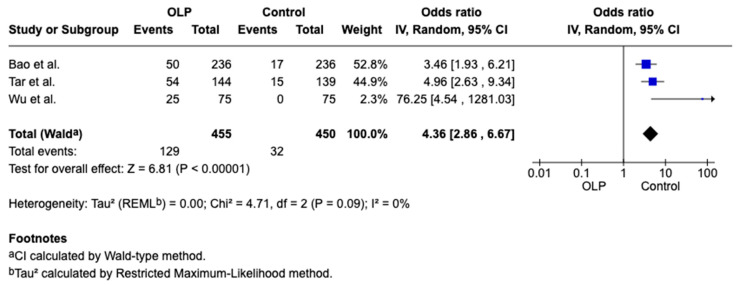
Forest plot showing the difference in iron deficiency prevalence between OLP patients and healthy controls [16,22,27].

Three articles [16,22,27] of the studies included in this work provided data linking iron deficiency with OLP; significant differences were observed between the two groups, as shown in Figure 4.

Forest plots were also generated to illustrate the concentrations of the various parameters reported in the studies, with the aim of comparing values between patients with OLP and healthy subjects. Initially, the units were unified to allow for direct comparability of the values across different studies. When studies did not provide mean and standard deviation data, the formula by Hozo et al. [28] was applied to estimate these values, allowing for comparable data to be included in the graphical analysis. The study of Sahebjamee et al. [24] not be included in the meta-analysis because it did not report the quantitative data required (mean and standard deviation or individual values). Instead, the results were presented only as two categorical, which prevents their integration into the quantitative analysis.

#### 3.4.1. Hemoglobin

**Figure 5:**
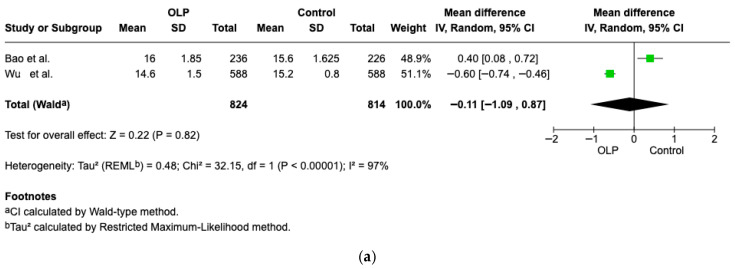

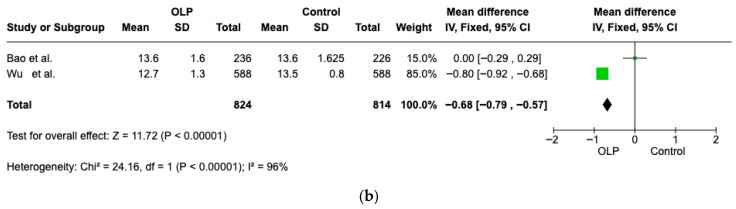
(**a**) Forest plot of the difference in Hb concentration between OLP male patients and healthy controls [16,23]. (**b**) Forest plot of the difference in Hb concentration between OLP female patients and healthy controls [16,23].

Hb levels were assessed in two studies [16,23], with results stratified by sex in both instances. In men, the difference was not statistically significant, although patients with OLP exhibited slightly lower Hb concentrations (Weighted Mean Difference (WMD): −0.11, 95% CI: −1.09 to 0.87, *p* = 0.82; heterogeneity 97%, *p* < 0.00001 Figure 5a. In women, however, a significant difference was observed between the two groups (Weighted Mean Difference (WMD): −0.68, 95% CI: −0.79 to −0.57, *p* < 0.00001; heterogeneity 97%, *p* < 0.00001 Figure 5b.

#### 3.4.2. Folic Acid

**Figure 6:**
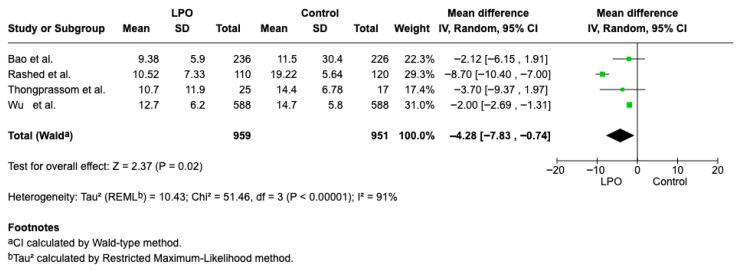
Forest plot of the difference in Folic Acid concentration between OLP patients and healthy controls [10,16,23,26].

Four studies [10,16,23,26] reported the necessary quantitative data and were included in the meta-analysis. The difference in concentration of acid folic in healthy patients and patients with OLP is statistically significant (Weighted Mean Difference (WMD): −4.28, 95% CI: −7.83 to −0.74, *p* = 0.02 and heterogeneity 91% *p* < 0.00001 Figure 6.

#### 3.4.3. Vitamin B12

**Figure 7:**
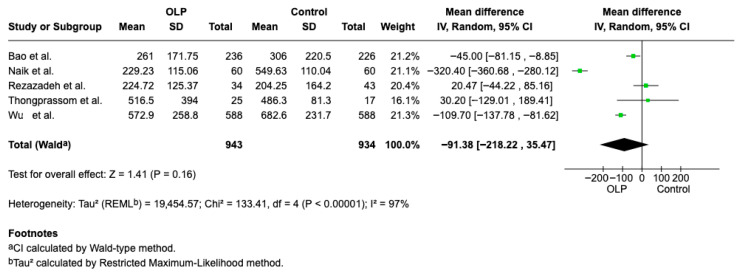
Forest plot of the difference in Vitamin B12 concentration between OLP patients and healthy controls [10,11,16,23,25].

Quantitative data necessary for the analysis were reported in five studies [10,11,16,23,25]. The difference in concentration of Vitamin B12 in healthy patients and patients with OLP is not statistically significant (Weighted Mean Difference (WMD): −91.38, 95% CI: −218.22 to 35.47, *p* = 0.16 and heterogeneity 97% *p* < 0.00001). The data indicate a tendency for patients with OLP to exhibit lower vitamin B12 concentrations than healthy individuals Figure 7.

#### 3.4.4. Iron

**Figure 8:**
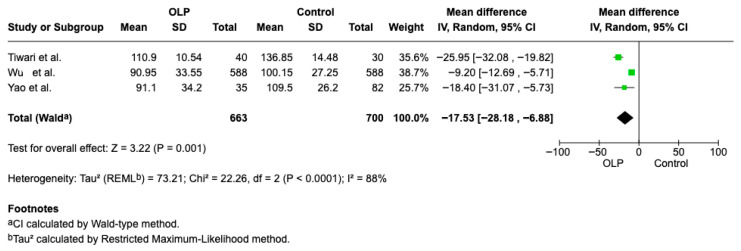
Forest plot of the difference in Iron concentration between OLP patients and healthy controls [20,21,23].

Blood iron concentration data were available from three studies [20,21,23], which were included in the comparative analysis. The difference in concentration of Iron in healthy patients and patients with OLP is statistically significant (Weighted Mean Difference (WMD): −17.53, 95% CI: −28.18 to −6.8, *p* = 0.0011 and heterogeneity 88% *p* < 0.00001 Figure 8.

## 4. Discussion

The present systematic review aims to synthesize the available evidence regarding the relationship between anemia and OLP, with a particular focus on hematological and micronutrient parameters commonly assessed in clinical practice. Specifically, this review examines studies that have evaluated blood-based parameters related to anemia, including hemoglobin, hematocrit, serum iron, ferritin, vitamin B12, folate, and homocysteine levels, in patients with OLP, in order to clarify the current state of evidence on the potential association between these conditions.

According to a review by Gholizadeh and Sheykhbahaei [29] examining the micronutrient profile of patients with OLP, it was found that individuals with OLP exhibited lower levels of several micronutrients, including vitamin A, vitamin B12, folate, and ferritin, compared with healthy controls. The investigators also found that, in most of the included studies, supplementation of these deficiencies led to improvements in clinical symptoms (such as burning sensation, pain, and lesion size) as well as in the levels of inflammatory markers or indicators [29].

Micronutrients play a fundamental role in preserving the body’s biodynamic balance. Imbalances can contribute to the onset and progression of numerous diseases. When micronutrient levels are inadequate, the host’s natural defense mechanisms become dysregulated, leading to impaired immune responses that affect physical barriers of the skin and mucosa, as well as cellular and humoral immunity. These micronutrients play a key role in tissue regeneration and in limiting oxidative stress–induced damage [30]. Insufficient levels may further impair essential host defense processes, including membrane integrity, apoptotic regulation, metabolic functions, and enzymatic activity [31]. Numerous studies have reported that patients with various inflammatory conditions, like OLP, frequently exhibit decreased levels of micronutrients such as iron, zinc, calcium, vitamin B12, folic acid, and vitamins C, D, and E [30,32].

According to established criteria, anemia is characterized by hemoglobin concentrations below 13.0 g/dL in men and 12.0 g/dL in women [1]. Sex differences in adult Hb levels are mainly driven by sex hormones: androgens, like testosterone, stimulate red blood cell production, while estrogens have a weaker effect. This results in higher Hb levels in men compared to women [33]. The absence of a significant difference in Hb levels in men with OLP, contrasted with a significant difference observed in women, can be explained by etiopathogenic mechanisms related to chronic inflammation and iron deficiency. In women with OLP, menstrual blood loss and lower iron stores make them more susceptible to iron deficiency and anemia, which may result in greater variability in Hb levels compared to men [34]. Additionally, the persistent inflammation characteristic of OLP can exacerbate these hematologic deficiencies, particularly in women who already have lower serum iron levels [35]. Studies have shown that female patients with OLP exhibit significantly higher rates of Hb and iron deficiency than male patients. Furthermore, hormonal differences may also play a role: androgens promote erythropoiesis and higher Hb levels, whereas female hormones can upregulate hepcidin, inhibiting intestinal iron absorption [34]. Taken together, these factors suggest that Hb is a more sensitive marker of disease-related iron deficiency in women with OLP than in men, whose higher baseline levels and greater iron reserves may mask modest relative changes. However, the observed difference in Hb levels in women was largely driven by a single large study [22] and should therefore be interpreted with caution. The current evidence does not support firm conclusions regarding a sex-specific pathophysiological difference in the Hb–OLP relationship. It is likely that iron deficiency, rather than anemia itself, acts as the main mediator of this association.

Given its essential role in cellular differentiation and proliferation, iron deficiency may contribute to epithelial thinning, mucosal inflammation, and atrophic changes. These alterations could increase mucosal permeability and vulnerability to environmental factors, potentially impairing local immune responses [29,31]. In addition, iron plays a role in collagen synthesis, suggesting that marked reductions in serum iron levels may impair oral mucosal integrity by disrupting extracellular matrix maintenance [20]. The study of Chang et al. [36] found that patients with EOLP, both those positive for anti-gastric parietal cell antibodies and those without antibodies, exhibited significantly lower levels of vitamin B12 and, in women, serum iron compared to individuals without EOLP [36]. Moreover, another study [20] indicates that iron deficiency may be linked to oral carcinogenesis, as lower iron levels were associated with both PMDs and malignant lesions, implying that alterations in this essential trace element could contribute to the progression of oral mucosal lesions toward malignancy [20]. The available evidence indicates significantly lower iron levels were observed in patients with OLP. Authors have proposed iron supplementation as an adjuvant to corticosteroid therapy, which has resulted in a significant improvement of the lesions [22].

Vitamin B12 plays a critical role in maintaining oral mucosal health by supporting DNA synthesis and the continuous renewal of the epithelium, thereby preserving structural integrity and promoting effective tissue repair. Its involvement in cellular metabolism and immune cell differentiation contributes to a resilient mucosal barrier capable of responding appropriately to inflammatory processes. Consequently, inadequate vitamin B12 levels have been associated with several oral mucosal disorders, including OLP and other conditions like atrophic glossitis or recurrent aphthous stomatitis [37]. The study of Lin et al. [38] demonstrated that supplementation with vitamin B12, either alone or in combination with levamisole, significantly reduced serum gastric parietal cell antibodies in OLP patients positive for these autoantibodies, suggesting a potentially beneficial immunomodulatory effect. Similarly, Naik et al. [11] reported that a substantial proportion of symptomatic OLP patients exhibit markedly reduced serum vitamin B12 levels, indicating that B12 deficiency may contribute to mucosal inflammation and the chronicity of OLP lesions. Furthermore, Wu et al. [39] reported OLP patients with vitamin B12 deficiency show higher frequencies of anemia, macrocytosis, hyperhomocysteinemia, and GPCA positivity compared with healthy controls, underscoring the association between B12 deficiency, autoimmune gastric dysfunction, and OLP pathophysiology. These findings support that correction of the deficiency may facilitate clinical improvement and lesion resolution in affected patients [37].

Vitamin B12 and folate are often assessed together because a decrease in serum B12 impairs folate metabolism, leading to reduced folate levels [29]. These two micronutrients are closely linked through one-carbon metabolism, essential for DNA synthesis and methylation. B12 acts as a cofactor for methionine synthase, converting homocysteine to methionine using 5-methyltetrahydrofolate as a substrate. B12 deficiency blocks this reaction, “trapping” folate in its methylated form and preventing its use in nucleotide synthesis and DNA repair, leading to a functional folate deficiency with impacts on cell proliferation, genome integrity, and hematologic and neurological health [40]. Folic acid plays a crucial role in regulating T cell–mediated apoptosis. A deficiency in folate can lead to a reduction in circulating T lymphocytes, limit their proliferative capacity, compromise innate immune responses, and reduce resistance to infections, particularly in older adults. Together with vitamin B12, folic acid is vital for proper cell growth and overall metabolic processes [29]. The existing literature suggests that patients with OLP were found to have significantly lower folic acid levels compared to healthy individuals. The reviewed studies by Chen et al. [9] and Thongprasom et al. [10] highlight the potential therapeutic role of folic acid supplementation in patients with OLP. Thongprasom et al. [10] reported that a significant proportion of patients exhibited reduced erythrocyte folate levels and suggested that folic acid administration could help improve oral mucosal health and potentially alleviate symptoms. Similarly, Chen et al. [9] identified a significant association between folate deficiency and the presence of OLP, supporting the notion that folic acid supplementation may serve as a complementary therapeutic strategy in patients with folate deficiency, aiding in the restoration of normal cellular function and reducing disease-associated inflammation.

This review has several limitations that should be considered. First, many studies on OLP focus primarily on local treatment strategies, such as topical corticosteroids or immunomodulators, and often do not address the systemic aspects of the disease; despite evidence supporting the association of OLP with systemic conditions, including thyroid disorders or cardiovascular diseases. Consequently, the systemic aspects of the disease are often underexplored. As a result, relevant laboratory data, including hematologic and micronutrient profiles, are frequently not reported, limiting the ability to fully assess associations between OLP and anemia or other systemic deficiencies. There is a scarcity of studies evaluating micronutrient supplementation, such as iron, folic acid, or vitamin B12, as a therapeutic intervention for OLP. This limits the evidence available to support systemic treatment strategies and constrains conclusions regarding the potential benefits of correcting nutritional deficiencies in affected patients. Likewise, the included studies were observational and cross-sectional in design. Consequently, the temporal relationship between hematinic deficiencies and OLP cannot be determined. Therefore, causal associations between these conditions cannot be established. Finally, the high level of heterogeneity observed across the included studies represents an important limitation of this review. Such heterogeneity was expected and can be attributed to multiple factors, including differences in study populations (geographical origin, age, and sex distribution), variability in the clinical subtypes and severity of OLP, and methodological diversity among studies. Variations in study design, sample size, diagnostic criteria, laboratory assays, and reported outcome measures further complicate the interpretation and generalizability of the findings.

## 5. Conclusions

This systematic review and meta-analysis provide consistent evidence that OLP is frequently accompanied by systemic hematological and micronutrient alterations. Patients with OLP show a significantly higher prevalence of anemia and deficiencies in key hematinic factors, particularly iron and folic acid, with sex-specific differences observed in hemoglobin levels. These findings support the concept that OLP should not be regarded solely as a localized mucosal disorder, but rather as a chronic inflammatory condition influenced by the patient’s overall systemic, nutritional, and inflammatory status.

The observed associations between OLP and hematinic deficiencies suggest the existence of shared pathogenic mechanisms, including chronic inflammation, immune dysregulation, impaired epithelial turnover, and altered micronutrient metabolism. Such systemic disturbances may contribute not only to disease onset and persistence, but also to symptom severity, mucosal vulnerability, and potentially to malignant transformation risk.

From a clinical perspective, routine laboratory evaluation of hematological parameters and micronutrient status should be considered in patients with OLP in cases with persistent or refractory symptoms. Identification and correction of underlying deficiencies may represent a modifiable factor in disease management. In this context, micronutrient supplementation—when clinically indicated—may serve as a valuable adjunct to conventional anti-inflammatory or immunosuppressive therapies, contributing to improved mucosal healing and symptom control.

## Figures and Tables

**Table 1:** Characteristics of the included studies.

Author and Year	Study Groups Sex/Age (Median ± ds) [Range]	Parameter (Unit) *p* Value Median ± ds [Range]/ Parameter-*p* Value-n		Conclusions
Thongprasom et al. 2001 [10]	25 OLP	Folic acid (ng/mL) < 0.001 *		Patients with oral lesions and associated clinical manifestations may exhibit suboptimal folate levels despite a normal complete blood count. Therefore, evaluation of folate status is advisable, with supplementation considered when deficiency is identified or suspected.
	21 F–4 M	OLP: 10.7 [1.3–48.9]		
	49 ± 13.5 [21–74]	C: 14.4 [5.4–32.5]		
	17 C	Vit B12 (pg/mL) NS		
	12 F–5 M	OLP: 516.5 (199–1775)		
	35.4 ± 11.3 [21–60]	C: 486.3 [321–646]		
Sahebjamee et al. 2009 [24]	32 OLP	Folic acid (mg/mL) 0.49		Vit B12 deficiency is more common in patients with OLP, while folate deficiency is rare. Although a clear causal relationship was not established, B12 assessment is suggested in symptomatic cases, as vitamin deficiencies may contribute secondarily to the disease.
	28 F–4 M	<1.5: 1 OLP/0 C		
	-	>1.5: 31 OLP/16 C		
	15 C	Vit B12 (pg/mL) 0.799		
	11 F–4 M	<160: 8 OLP/2 C		
	-	>160: 2 OLP/14 C		
Wu et al. 2014 [22]	75 IDA 64 F–11 M 51.7 ± 14.1 [20–88] 150 C 128 F–22 M 52.4 ± 13.8 [21–89]	OLP: <0.001 * IDA: 25/C: 0 BS: <0.001 * IDA: 57/C: 0 DM: <0.001 * IDA: 37/C: 0	AG: <0.001 * IDA: 20/C: 0 RAU: <0.001 * IDA: 19/C: 0	Individuals with IDA showed a significantly higher prevalence of oral manifestations than healthy controls, most commonly oral burning, lingual varicosities, xerostomia, OLP, and atrophic glossitis. These patients also presented significantly lower Hb levels, RBC counts, MCV, Mentzer index, serum iron, and vit B12 concentrations.
Rashed et al. 2017 [26]	110 OLP 18 F–92 M 39.73 ± 11.84 120 C 24 F–96 H 41.13 ± 9.55	Folic acid μmol/mL < 0.001 * 10.52 ± 7.33 [3.02–28.3]		Patients with OLP have lower folate levels, particularly those with the MTHFR 677 TT polymorphism, which may affect homocysteine metabolism. This folate deficiency could increase cardiovascular risk and may be a predisposing factor for OLP.
Tiwari et al. 2016 [21]	40 PMDs 10 F–30 M 41.1 ± 12.14 30 C 13 F–17 M 33.26 ± 7.72	Iron μg/100 mL < 0.001 * PMD: 110.9 ± 10.54 C: 136.85 ± 14.48		In patients with PMD and OSCC, increased levels of CICs and trace elements such as copper have been reported, accompanied by reduced serum iron concentrations. These suggest a close involvement of trace elements and immune complexes in disease pathogenesis and progression.
Naik et al. 2020 [11]	60 OLP 38 F–22 M 60 C - Total population: 38.5	Vit B12 (pg/mL) 0 OLP: 229.23 ± 115.06 C: 549.63 ± 110.04		Reduced vit B12 levels have been observed in patients with OLP, suggesting a potential role for vit B12 supplementation in affected individuals.
Bao et al. 2020 [16]	236 OLP 41 M–195 F 51.70 ± 13.99 226 C 52 M–174 F 49.46 ± 17.26	Hb (g/dL) (M) 0.798 OLP: 160 ± 18.5 C: 156.16.25 (F) 0.845 OLP: 136 ± 16 C: 136 ± 14.25 Folic acid (ng/mL) < 0.001 * OLP: 9.38 ± 5.9 C: 11.5 ± 30.4	Vit B12 (ng/L) < 0.001 * OLP: 261 ± 171.75 C: 306.5 ± 220.5 Ferritin (ng/mL) (M) 0.629 OLP: 126.8 ± 94.85 C: 120.3 ± 130.38 (F) 0.458 OLP: 54.2 ± 101.8 C: 38.65 ± 65.62	A significant association has been identified between OLP and hematinic deficiencies. Routine monitoring of iron, folate, and vit B12 levels may therefore be warranted in patients with this condition.
Rezazadeh et al. 2021 [25]	34 OLP (Erosive and Non-erosive) 11 M–23 F 48.031 ± 11.57 43 C 18 M–25 F 48.744 ± 12.7	Vit A ppm 0.508 (E vs. NE) 0.419 E: 55:51 ± 38:84 NE: 63:70 ± 43:86 C: 72:31 ± 8:57 Vit B12 ppm 0.77 (E vs. NE) 0.168 E: 18:42 ± 7:33 NE: 14:74 ± 11:22 C: 15:07 ± 1:25		Although serum concentrations of vit A, C, and E were lower in patients with OLP compared with healthy controls, these differences did not reach statistical significance. Consequently, these vit may not play a major role in the pathogenesis of OLP.
Tar et al. 2025 [27]	144 OLP 35 M–109 F 54.56 ± 12.37 [23–80] 53 OL 18 M–35 F 56.91 ± 12.31 [29–91] 139 C 43 M–96 F 52.52 ± 14.06 [16–82]	Anemia NS OLP: 49/C: 24 Iron deficiency 0.00 * OLP: 54/C: 15 Vit B deficiency NS OLP: 8/C: 8 Desquamative gingivitis: NS OLP: 25/C: 0		Management of comorbidities, particularly iron deficiency, was associated with reduced severity and extent of OLP and oral lichenoid lesions. Normalization of iron status appeared to support epithelial repair, attenuate mucosal inflammation, and was linked to improved clinical outcomes with lower recurrence rates.
Wu et al. 2025 [23]	588 OLP 110 M–478 F 55.8 ± 14.1 [20–88] 588 C 110 M–478 F age range 56.0 ± 14.1 [20–89]	Iron (μg/dL) (M) 0.308 OLP: 99.1 ± 36.8 C: 103.6 ± 27.9 (F) < 0.001 * OLP: 82.8 ± 30.3 C: 96.7 ± 27.4 Hb (g/dl) (M) < 0.001 * OLP: 14.6 ± 1.5 C: 15.2 ± 0.8 (F) < 0.001 * OLP: 12.7 ± 1.3 C: 13.5 ± 0.8	Vit B12 (pg/dL) < 0.001 * OLP: 572.9 ± 258.8 C: 682.6 ± 231.7 Folic acid (ng/dL) < 0.001 * OLP: 12.7 ± 6.2 C: 14.7 ± 5.8 MCV (fL) < 0.001 * OLP: 88.4 ± 8.5 C: 90.4 ± 3.8	These results demonstrate that patients with OLP have a significantly higher prevalence of anemia and deficiencies in serum iron, vit B12, and folate compared with healthy controls.
Yao et al. 2025 [20]	237 RAU 46 M–54 F 44 [18–70] 35 OLP 15 M–20 F 53 [36–70] 67 AG 15 M–52 F 55 [36–74] 35 BMS 10 M–25 F 58 [44–72] 82 Control 36 M–46 F 59 [50–68]	Iron (μmol/L) < 0.001 * OLP: 16.32 ± 6.12 C: 19.62 ± 4.70 Iron saturation (%) 0.003 * OLP: 30.17 ± 11.57 C: 32.84 ± 7.84		Several biomarkers, including B vit, serum iron, and zinc, were significantly associated with these conditions. Assessment of specific vit and micronutrient levels may therefore support the diagnosis and clinical management of oral mucosal lesions.

* Statistically significant. OLP: Oral Lichen Planus; C: Control; Hb: Hemoglobin; M: Male, F: Female; E: Erosive; NE: Non-Erosive; Vit: Vitamin; NS: No Significant; CICs: circulating immune complexes; PMDs: Potentially Malignant Disorders (moderate and severe dysplasia of leukoplakia, erosive OLP and clinically diagnosed oral submucous fibrosis); IDA: Iron Deficiency Anemia; MCV: Mean Corpuscular Volume; RBC: Red Blood Cells, RAU: Recurrent Aphthous Ulcers; AG: Atrophic Glossitis; OL: Oral leukoplakia; OSCC: Oral Squamous Cell Carcinoma; BS: Burning sensation; DM: Dry mouth.

**Table 2:** Diagnostic criteria for OLP used by each study.

Author and Year	Diagnostic Criteria
Thongprasom et al. 2001 [10]	Clinical criteria: Bilateral and usually symmetrical oral lesions presence of reticular white striae Wickham striae typical clinical forms including reticular atrophic erosive ulcerative or bullous involvement of buccal mucosa tongue and gingiva chronic clinical course. Histological criteria: Band-like lymphocytic inflammatory infiltrate at the epithelial–connective tissue interface hydropic degeneration of the basal cell layer presence of apoptotic keratinocytes Civatte bodies variable epithelial changes including hyperkeratosis or epithelial atrophy absence of epithelial dysplasia.
Sahebjamee et al. 2009 [24]	Clinical and histopathological criteria not explicitly specified.
Wu et al. 2014 [22]	Clinical criteria: radiating grayish-white Wickham striae, papules, plaques, either individually or in combination, and erosion or ulceration on the oral mucosa; a bilateral and symmetric distribution of OLP lesions on the oral mucosa. Histological criteria: hyperkeratosis or parakeratosis, a slightly acanthotic epithelium with liquefaction degeneration of basal epithelial cells, a prominent band-like lymphocytic infiltrate in the lamina propria, and the absence of epithelial dysplasia.
Rashed et al. 2017 [26]	Clinical and histopathological criteria not explicitly specified.
Tiwari et al. 2016 [21]	Clinical and histopathological criteria not explicitly specified
Naik et al. 2020 [11]	Clinical and histopathological criteria not explicitly specified
Bao et al. 2020 [16]	Clinical criteria: Bilateral and usually symmetrical oral lesions presence of reticular white striae Wickham striae typical clinical forms including reticular atrophic erosive ulcerative or bullous involvement of buccal mucosa tongue and gingiva chronic clinical course. Histological criteria: Band-like lymphocytic inflammatory infiltrate at the epithelial–connective tissue interface hydropic degeneration of the basal cell layer presence of apoptotic keratinocytes Civatte bodies variable epithelial changes including hyperkeratosis or epithelial atrophy absence of epithelial dysplasia.
Rezazadeh et al. 2021 [25]	Clinical and histopathological criteria not explicitly specified.
Tar et al. 2025 [27]	Clinical criteria: more or less symmetrical lesions and the presence of Wickham’s striae. The lesions can be categorized as reticular, popular, plaque-like (non-erosive, non-atrophic types), or atrophic, erosive, or bullous types (atrophic–erosive types). Histological criteria: included the presence of ribbon-like subepithelial lymphocyte infiltration, liquefaction degeneration (exocytosis), and hyperkeratosis with the thickening of the granular cell layer.
Wu et al. 2025 [23]	Clinical criteria: radiating grayish-white Wickham striae, papules, plaques, either individually or in combination, and erosion or ulceration on the oral mucosa; a bilateral and symmetric distribution of OLP lesions on the oral mucosa. Histological criteria: hyperkeratosis or parakeratosis, a slightly acanthotic epithelium with liquefaction degeneration of basal epithelial cells, a prominent band-like lymphocytic infiltrate in the lamina propria, and the absence of epithelial dysplasia.
Yao et al. 2025 [20]	Clinical criteria: Chronic oral lesions usually bilateral and symmetrical presence of reticular white striae possible atrophic erosive ulcerative or bullous forms common involvement of buccal mucosa tongue and gingiva chronic relapsing course. Histological criteria: Band like lymphocytic inflammatory infiltrate at the epithelial connective tissue interface basal cell layer degeneration presence of apoptotic keratinocytes Civatte bodies epithelial changes consistent with OLP absence of epithelial dysplasia.

**Table 3:** Summary of Evidence: NOS Scores and GRADE Levels of Recommendation.

Author/Year	Selection of Cases and Controls	Comparability of Cases and Controls	Ascertainment of Exposure	Conclusion	GRADE
Thongprasom et al. 2001 [10]	⋆⋆⋆⋆	⋆⋆	⋆⋆	High quality	2/B
Shahebjamee et al. 2009 [24]	⋆⋆⋆⋆	⋆⋆	⋆⋆⋆	High quality	2/B
Wu et al. 2014 [22]	⋆⋆⋆⋆	⋆⋆	⋆⋆	High quality	2/B
Tiwari et al. 2016 [21]	⋆⋆⋆⋆	⋆⋆	⋆⋆	High quality	2/B
Rashed et al. 2017 [26]	⋆⋆⋆⋆	⋆⋆	⋆⋆⋆	High quality	2/B
Naik et al. 2020 [11]	⋆⋆⋆⋆	⋆⋆	⋆⋆⋆	High quality	2/B
Bao et al. 2020 [16]	⋆⋆⋆⋆	⋆⋆	⋆⋆⋆	High quality	2/B
Rezazadeh et al. 2021 [25]	⋆⋆⋆⋆	⋆	⋆⋆⋆	High quality	2/B
Wu et al. 2025 [23]	⋆⋆⋆⋆	⋆⋆	⋆⋆	High quality	2/B
Yao et al. 2025 [20]	⋆⋆⋆	⋆⋆	⋆	Moderate quality	3/C
Tar et al. 2025 [27]	⋆⋆⋆⋆	⋆	⋆⋆	High quality	2/B

Stars indicate study quality according to the Newcastle–Ottawa Scale: Selection (max 4 stars), Comparability (max 2 stars), Exposure/Outcome (max 3 stars). More stars = lower risk of bias.

## Data Availability

The original contributions presented in this study are included in the article. Further inquiries can be directed to the corresponding authors.

## Supplementary Materials

The following supporting information can be downloaded at: https://www.mdpi.com/article/10.3390/jcm15020581/s1, Preferred Reporting Items for Systematic reviews and Meta-Analyses extension for Scoping Reviews (PRISMA-ScR) Checklist [41].

## Abbreviations

The following abbreviations are used in this manuscript:

NOS	Newcastle-Ottawa
GRADE	Grades of Recommendation, Assessment, Development and Evaluation
WHO	World Health Organization
Hb	Hemoglobin
EOLP	Erosive Oral Lichen Planus
OLP	Oral Lichen Planus
M	Male
F	Female
E	Erosive
NE	No Erosive
Vit	Vitamin
NS	Non significant
CICs	Circulating Inmuno Complexes
PMDs	Potencially Malignant Disorders
IDA	Iron Definciency Anemia
MCV	Mean Corspuscular Volume
RCB	Red Blood Cells
RAU	Recurrent Aphtous Ulcers
AG	Atrophic Glossitis
OL	Oral Leukoplakia
OSCC	Oral Squamous Cell Carcinoma
BS	Burning Sensation
DM	Dry Mouth

## References

[1] Cappellini M.D., Motta I. (2015). Anemia in clinical practice—Definition and classification: Does hemoglobin change with aging?. Semin. Hematol..

[2] Chaparro C.M., Suchdev P.S. (2019). Anemia epidemiology, pathophysiology, and etiology in low- and middle-income countries. Ann. N. Y. Acad. Sci..

[3] Fernández-Bañares F., Monzón H., Forné M. (2009). A short review of malabsorption and anemia. World J. Gastroenterol..

[4] Dahlerup J.F., Eivindson M., Jacobsen B.A., Jensen N.M., Jørgensen S.P., Laursen S.B., Rasmussen M., Nathan T. (2015). Diagnosis and treatment of unexplained anemia with iron deficiency without overt bleeding. Dan. Med. J..

[5] Sugerman P.B., Savage N.W., Walsh L.J., Zhao Z.Z., Zhou X.J., Khan A., Seymour G.J., Bigby M. (2002). The pathogenesis of oral lichen planus. Crit. Rev. Oral Biol. Med..

[6] Eisen D. (2003). The clinical features, malignant potential, and treatment of oral lichen planus. Periodontol. 2000.

[7] Arias-Santiago S., Buendía-Eisman A., Aneiros-Fernández J., Girón-Prieto M.S., Gutiérrez-Salmerón M.T., Mellado V.G., Naranjo-Sintes R. (2011). Cardiovascular risk factors in patients with lichen planus. Am. J. Med..

[8] Scully C., Carrozzo M. (2008). Oral lichen planus: A review. Br. J. Oral Maxillofac. Surg..

[9] Chen H.M., Wang Y.P., Chang J.Y.F., Wu Y.C., Cheng S.J., Sun A. (2015). Significant association of deficiencies of hemoglobin, iron, folic acid, and vitamin B12 and high homocysteine level with oral lichen planus. J. Formos. Med. Assoc..

[10] Thongprasom K., Youngnak P., Aneksuk V. (2001). Folate and vitamin B12 levels in patients with oral lichen planus, stomatitis or glossitis. Southeast Asian J. Trop. Med. Public Health.

[11] Naik S.R., Gupta P., Khaitan T., Shukla A.K. (2020). Reduced levels of serum vitamin B12 in symptomatic cases of oral lichen planus: A cross-sectional study. J. Oral Biol. Craniofacial Res..

[12] Kleier C., Werkmeister R., Joos U. (1998). Zink- und Vitamin-A-Mangel bei Mundschleimhauterkrankungen. Mund Kiefer Gesichtschir..

[13] Di Vincenzo F., Del Gaudio A., Petito V., Lopetuso L.R., Scaldaferri F. (2024). Gut microbiota, intestinal permeability, and systemic inflammation: A narrative review. Intern. Emerg. Med..

[14] Wacka E., Nicikowski J., Jarmuzek P., Zembron-Lacny A. (2024). Anemia and its connections to inflammation in older adults: A review. J. Clin. Med..

[15] Mutafchieva M.Z., Draganova M.N., Tomov G.T. (2025). Molecular markers in oral lichen planus—Insight into pathogenesis. Head Neck Pathol..

[16] Bao Z.X., Yang X.W., Shi J., Wang Y.F. (2020). The profile of hematinic deficiencies in patients with oral lichen planus: A case-control study. BMC Oral Health.

[17] Page M.J., McKenzie J.E., Bossuyt P.M., Boutron I., Hoffmann T.C., Mulrow C.D., Shamseer L., Tetzlaff J.M., Akl E.A., Brennan S.E. (2021). The PRISMA 2020 statement: An updated guideline for reporting systematic reviews. BMJ.

[18] Wells G., Shea B., O’Connell D., Robertson J., Peterson J., Welch V., Losos M., Tugwell P. (2011). The Newcastle–Ottawa Scale for Assessing the Quality of Nonrandomized Studies in Meta-Analysis.

[19] Brożek J.L., Akl E.A., Alonso-Coello P., Lang D., Jaeschke R., Williams J.W., Phillips B., Lelgemann M., Lethaby A., Bousquet J. (2009). Grading quality of evidence and strength of recommendations in clinical practice guidelines: Part 1. Allergy.

[20] Yao H., Cao Z., Huang L., Pan H., Xu X., Sun F., Ding X., Wu W. (2025). Application of machine learning for the analysis of peripheral blood biomarkers in oral mucosal diseases: A cross-sectional study. BMC Oral Health.

[21] Tiwari R., David C.M., Mahesh D.R., Sambargi U., Rashmi K.J., Benakanal P. (2016). Assessment of serum copper, iron and immune complexes in potentially malignant disorders and oral cancer. Braz. Oral Res..

[22] Wu Y.C., Wang Y.P., Chang J.Y.F., Cheng S.J., Chen H.M., Sun A. (2014). Oral manifestations and blood profile in patients with iron deficiency anemia. J. Formos. Med. Assoc..

[23] Wu Y.H., Chang J.Y.F., Lee Y.P., Wang Y.P., Sun A., Chiang C.P. (2025). Anemia, hematinic deficiencies, hyperhomocysteinemia, and serum gastric parietal cell antibody positivity in 588 patients with oral lichen planus. J. Dent. Sci..

[24] Sahebjamee M. (2009). Assessment of serum vitamin B12 and folic acid in patients with oral lichen planus: A case-control study. Shiraz Univ. Dent. J..

[25] Rezazadeh F., Haghighat S. (2021). Serum vitamin profile in oral lichen planus patients in Southwest of Iran. BioMed Res. Int..

[26] Rashed L., Abdel Hay R., AlKaffas M., Ali S., Kadry D., Abdallah S. (2017). MTHFR 677 gene polymorphism, cardiovascular risk and lichen planus. J. Oral Pathol. Med..

[27] Tar I., Szarka K., Martos R., Kiss C., Márton I. (2025). Effects of comorbid disease improvement on oral lichen planus (OLP) and oral leukoplakia (OL) lesions: A retrospective longitudinal study. J. Clin. Med..

[28] Hozo S.P., Djulbegovic B., Hozo I. (2005). Estimating the mean and variance from the median, range, and the size of a sample. BMC Med. Res. Methodol..

[29] Gholizadeh N., Sheykhbahaei N. (2021). Micronutrients profile in oral lichen planus: A review. Biol. Trace Elem. Res..

[30] Aboushousha A., Kamal Y., Ali S. (2025). Supplementary zinc and vitamin D in management of symptomatic oral lichen planus: A three-arm randomized clinical trial. BMC Oral Health.

[31] Maggini S., Wintergerst E.S., Beveridge S., Hornig D.H. (2007). Selected vitamins and trace elements support immune function. Br. J. Nutr..

[32] See J.K.L., Liu X., Canfora F., Moore C., McCullough M., Yap T., Paolini R., Celentano A. (2023). The role of vitamins in oral potentially malignant disorders and oral cancer: A systematic review. J. Pers. Med..

[33] Murphy W.G. (2014). The sex difference in haemoglobin levels in adults—Mechanisms, causes and consequences. Blood Rev..

[34] Chang J.Y.F., Wu Y.H., Lee Y.P., Wang Y.P., Sun A., Chiang C.P. (2025). Anemia, hematinic deficiencies, and hyperhomocysteinemia in male and female oral lichen planus patients. J. Dent. Sci..

[35] Chang J.Y.F., Wu Y.H., Lee Y.P., Wang Y.P., Sun A., Chiang C.P. (2025). Anemia, hematinic deficiencies, hyperhomocysteinemia, and gastric parietal cell antibody positivity in oral lichen planus patients with iron deficiency. J. Dent. Sci..

[36] Chang J.Y.F., Wang Y.P., Wu Y.H., Su Y.X., Tu Y.K., Sun A. (2018). Hematinic deficiencies and anemia statuses in gastric parietal cell antibody-positive or autoantibody-negative erosive oral lichen planus patients. J. Formos. Med. Assoc..

[37] Putri N.T., Nur’aeny N., Sufiawati I. (2024). Vitamin B12 levels in patients with oral mucosal diseases: A systematic review. Clin. Nutr. Open Sci..

[38] Lin H., Wang Y., Chia J., Chiang C., Sun A. (2011). Modulation of serum gastric parietal cell antibody level by levamisole and vitamin B12 in oral lichen planus. Oral Dis..

[39] Wu Y.H., Chang J.Y.F., Lee Y.P., Wang Y.P., Sun A., Chiang C.P. (2025). Anemia, hematinic deficiencies, hyperhomocysteinemia, and gastric parietal cell antibody positivity in oral lichen planus patients with vitamin B12 deficiency. J. Dent. Sci..

[40] Brito A., Hertrampf E., Olivares M., Gaitán D., Sánchez H., Allen L.H., Uauy R. (2012). Folatos y vitamina B12 en la salud humana. Rev. Méd. Chile.

[41] Tricco A.C., Lillie E., Zarin W., O’Brien K.K., Colquhoun H., Levac D., Moher D., Peters M.D.J., Horsley T., Weeks L. (2018). PRISMA Extension for Scoping Reviews (PRISMAScR): Checklist and Explanation. Ann. Intern. Med..

